# First Whole Transcriptome RNAseq on CHD8 Haploinsufficient Patient and Meta-Analyses Across Cellular Models Uncovers Likely Key Pathophysiological Target Genes

**DOI:** 10.7759/cureus.11571

**Published:** 2020-11-19

**Authors:** Heba Yasin, Robert Stowe, Chi Kin Wong, Puthen Veettil Jithesh, Farah R Zahir

**Affiliations:** 1 Life Science, Hamad Bin Khalifa University, Doha, QAT; 2 Psychiatry and Neurology, University of British Columbia, Vancouver, CAN; 3 Medical Genetics, University of British Columbia, Vancouver, CAN

**Keywords:** chd8, intellectual disability, neurodevelopmental disorders, autism spectrum disorder, rnaseq

## Abstract

In 2019, we confirmed that the haploinsufficiency of CHD8 does indeed cause the novel syndromic neurodevelopmental disease we first discovered a dozen years before. Here, we report the first whole transcriptome RNAseq gene expression profiling for a patient with this new syndrome, as a preliminary exploration of potential pathophysiological mechanisms. We compared our patient transcriptome profile with that of all publicly available RNAseq datasets from human cellular models including neuronal progenitor cells, neurons and organoids. We compared differential gene expression profiles overall and conducted phenotype-informed data filtration based on the characteristic syndrome presentation. We found that concordance among differential gene expression profiles was poor across all datasets. Nevertheless, remarkably, we show that the patient blood differential gene expression profile most resembled that of the neuronal cell model, a finding that encourages further transcriptome profiling using patient blood samples. In addition, our custom phenotype-informed analyses yielded important, differentially expressed syndrome pathophysiology target genes. Finally, we note that genes dysregulated due to CHD8 heterozygous deletion are linked to known neurological as well as oncological pathways.

## Introduction

In 2007, we first reported that the haploinsufficiency of CHD8 likely is syndromic for neurodevelopmental disease (ND) [1]. In the dozen years that followed, several others also published reports of CHD8 causation for neurodevelopmental conditions, including developmental delay (DD), intellectual disability (ID) and autism spectrum disorder (ASD) (see [2] for a review of these studies). Recently, we conducted a comprehensive review of all reported patients [2], and established that the haploinsufficiency of CHD8 is indeed causative for a novel neurodevelopmental syndrome, which we propose be called Zahir-Friedman syndrome (ZFS) [3]. The syndrome presentation includes DD and/or ID and/or ASD and macrocephaly, speech delay, gastrointestinal (GI) problems, sleep problems, and skeletal and motor problems as characteristic, along with a distinguishing, if subtle, facial gestalt [2].

CHD8 is a chromatin remodeling gene with wide effects on gene regulation and transcription. Following the reports of CHD8+/- as causative for ND [1], a bourgeoning interest in the gene led to the publication of key functional studies; important among them, transcriptional exploration in human cellular models - induced pluripotent stem cell derived-neuronal progenitor cells (NPC) [4-6], neurons [6] and cerebral organoids [7]. Yet, none of these studies explored the specific phenotypic outcomes presented by the syndrome in their analyses.

Here, we present the first whole transcriptome RNAseq analyses of a peripheral blood mononuclear cell (PBMC) sample for a ZFS patient. We chose patient Vancouver 4444, who has the smallest copy number variant (CNV) overlapping the critical region [2] and compared whole-genome expression profiles for him versus his normal age and sex-matched sibling in triplicate for this preliminary exploratory study. We have previously verified that this patient’s CNV results in halved CHD8 gene expression [2]. In order to meaningfully assess the patient blood-derived transcriptome, we obtained complete whole transcriptome datasets from all published human induced pluripotent stem cells (iPSC)-derived CHD8+/- model studies available at the time and compared our patient’s data to them [4-7]. We scrutinize all datasets using a custom analysis based on syndrome pathophysiology. By doing so, we are able to highlight key downstream targets of CHD8 that potentially play a role in the development of this syndrome.

## Materials and methods

Patient RNAseq

Whole blood was drawn into ethylenediaminetetraacetic acid (EDTA) tubes from the patient and his normal brother (age and sex-matched control). PBMCs were isolated and total RNA was extracted using TRIzol and RNeasy kits (Qiagen; Hilden, Germany), as previously described [2]. Whole transcriptome RNAseq on the patient and his normal brother was performed in triplicate by the Biomedical Research Center at the University of British Columbia (BRC-UBC) sequencing platform as per their standard protocol. Samples were quality checked using the Agilent 2100 bioanalyzer (Agilent Technologies, Santa Clara, CA). Samples were then prepped following the standard protocol for the NEBnext Ultra II Standard mRNA kits (New England Biolabs, Ipswich, MA). Sequencing was performed on the Illumina NextSeq 500 (San Diego, California) with paired-end 42bp X 42bp reads. Bioinformatic data analysis was generated by the BRC-UBC as per their standard analysis pipeline: de-multiplexed read sequences were aligned to the reference sequence using STAR (http://code.google.com/p/rna-star/). Cufflinks were used to estimate differential expression. RNAseq data were reposited at the European Nucleotide Archive and available at https://www.ebi.ac.uk/ega/datasets/EGAD00001006006.

Selection of publicly available RNAseq data

We selected five publicly available human cellular whole transcriptome datasets, comprising a total of 26 samples and 18 control datasets from five separate RNAseq datasets from four publications [4-7]. Appendix 1 tabulates laboratory methods and bioinformatics pipelines for the studies.

Selection of differentially expressed genes (DEGs)

DEGs were selected based on q-values generated by the Benjamini-Hochberg correction using a uniform threshold of q<0.05 for each study.

Obtaining phenotype seed genes (PSGs)

The Phenolyzer (http://phenolyzer.wglab.org/, accessed January-May 2018) was used to generate a list of candidate genes termed a ‘phenotype seed gene (PSG) list’ for each of the 12 characteristic syndrome phenotypes. PSGs were generated using the search terms, as described in Appendix 2. PSGs obtained per phenotype are given in Appendix 3. 

Pathway analyses

Pathway analyses were conducted for DEGs using Ingenuity Pathway Analysis (IPA®) from Ingenuity (http://www.ingenuity.com) as described in Appendix 2.

## Results

Patient RNAseq results

Close to Half of the Expressed Genes Are Differentially Expressed in the Patient

**Figure 1:**
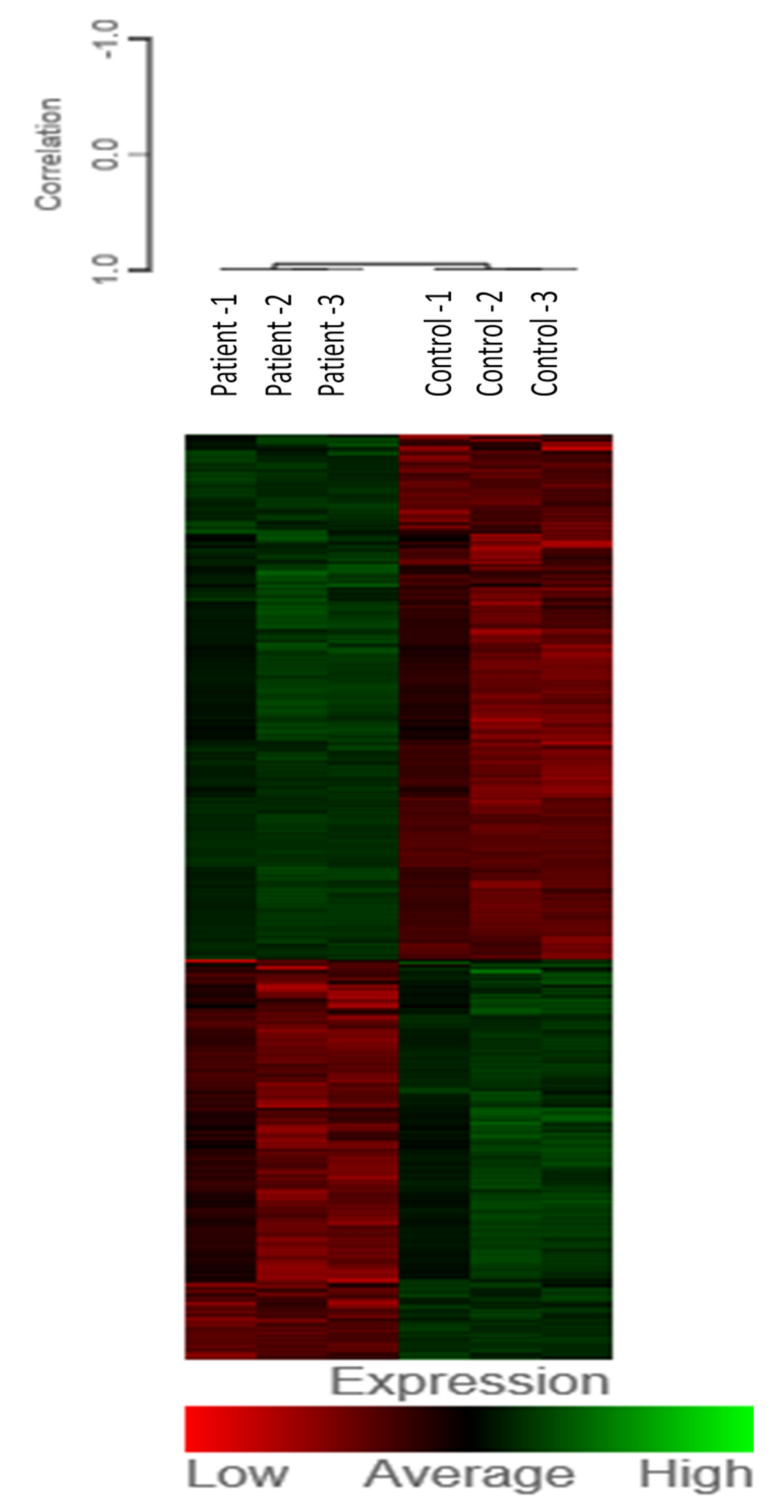
Whole transcriptome RNAseq heatmap for differential expression between patient and control samples

Patient RNA sequencing detected 13,002 genes and 21,493 transcripts (Appendix 3 contains tables of expression levels for all detected genes). Differential gene expression analysis between patient and control revealed 5,388 significant DEGs (41% of all detected genes) at Benjamini-Hochberg q<0.05, separating patient and control samples distinctively (Figure 1).

Slightly More Genes Were Upregulated (52%) Than Downregulated (48%)

**Figure 2:**
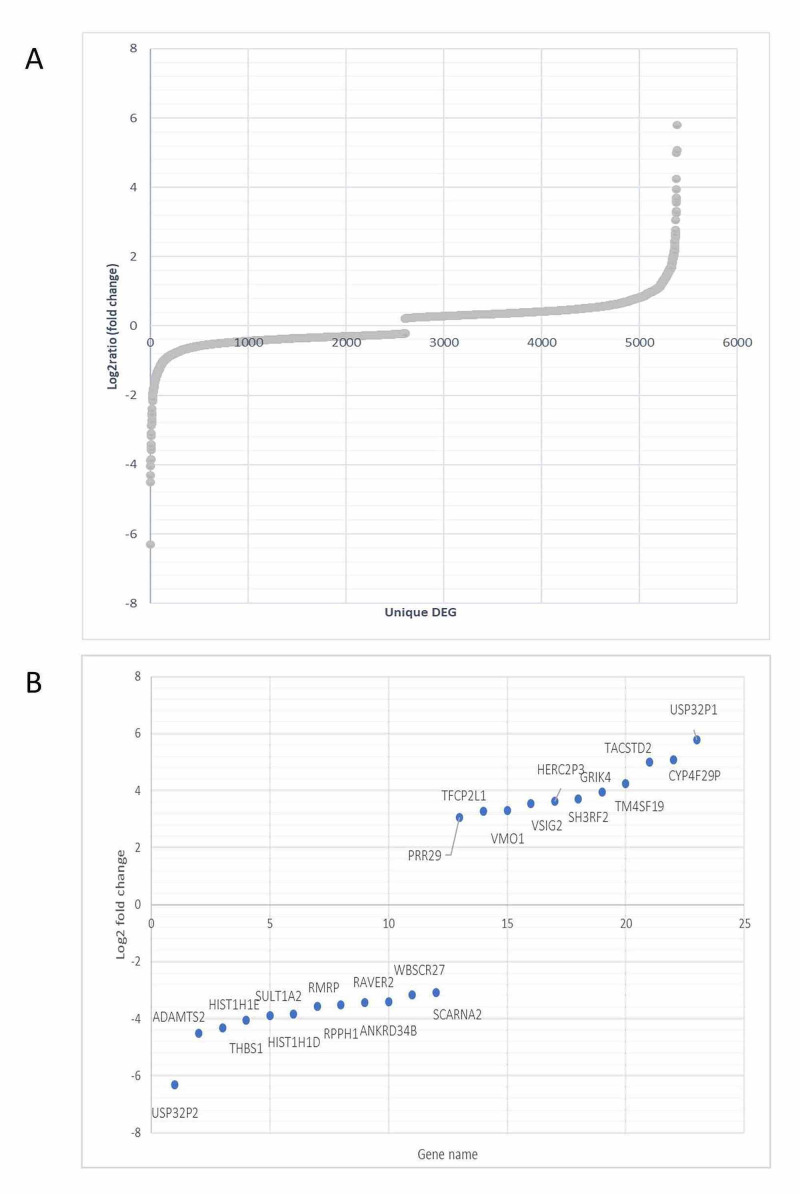
A – log2 fold change scatter plot showing differential gene expression between patient and control across all 5,388 detected transcripts. B – 23 genes that showed a log2 fold change ± 3 between patient and control

Of the 5,388 DEGs in the patient, 386 had a fold change greater or equal to ± 1, 64 had a fold change greater or equal to ± 2, and 23 had a fold change ± 3 (Figures 2A-2B and Table 1). Both the most upregulated and most downregulated DEGs in the patient are pseudogenes belonging to the ubiquitin peptidase family. Only three of the 23 genes in Table 1 appear as DEGs in any of the other datasets we analyzed (Appendix 3). 

**Table 1:** DEGs in patients that are expressed with a log2 fold change of either <= -3 or <=3 DEGs: differentially expressed genes

GENE	LOG2 (FOLD_CHANGE)	P_VALUE	Q_VALUE	REGULATION IN PATIENT	OTHER DATASETS GENE IS A DEG IN
USP32P2	-6.312	5.00E-05	0.000316967	down	None
ADAMTS2	-4.49852	0.00025	0.00129193	down	Wang_Neurons
HIST1H1E	-4.31541	5.00E-05	0.000316967	down	None
THBS1	-4.04863	5.00E-05	0.000316967	down	Wilkinson, Wang_NPC and Wang_Neurons
SULT1A2	-3.87789	0.00655	0.019417	down	None
HIST1H1D	-3.83365	0.00015	0.000841735	down	Wilkinson
RMRP	-3.57236	5.00E-05	0.000316967	down	None
RPPH1	-3.5139	5.00E-05	0.000316967	down	None
RAVER2	-3.42626	0.0004	0.00193051	down	None
ANKRD34B	-3.41265	0.00035	0.00172049	down	None
WBSCR27	-3.16821	0.0077	0.0223024	down	None
SCARNA2	-3.09253	5.00E-05	0.000316967	down	None
PRR29	3.06369	0.00145	0.00555477	up	None
TFCP2L1	3.26474	5.00E-05	0.000316967	up	None
VMO1	3.3094	5.00E-05	0.000316967	up	None
VSIG2	3.55333	0.0196	0.0477048	up	None
HERC2P3	3.63294	5.00E-05	0.000316967	up	None
SH3RF2	3.70726	0.00385	0.0124584	up	None
GRIK4	3.95099	0.0002	0.00107189	up	None
TM4SF19	4.23945	0.00115	0.00461064	up	None
TACSTD2	4.99907	0.0026	0.00902676	up	None
CYP4F29P	5.07494	0.0001	0.000592077	up	None
USP32P1	5.78815	5.00E-05	0.000316967	up	None

The Ubiquitination Proteasome Pathway (UPP) is the Most Differentially Expressed Pathway in Patients

IPA was used to explore differentially expressed canonical pathways and networks in the patient by inputting all patient DEGs. Of the 5,388 DEGs, all mapped in IPA, except for 11 uncharacterized, long, intergenic, non-protein-coding RNAs, which were unrecognized. Table 2 lists the top 10 differentially expressed pathways in the patient, with the UPP shown to be the most differentially expressed. M-TOR signaling is the next most perturbed. Among affected networks, the most affected is “Developmental Disorder, Hereditary Disorder, Neurological Disease” (Appendix 4).

**Table 2:** Top 10 differentially expressed pathways in the patient versus normal control

Ingenuity Canonical Pathways	-log (p-value)
Protein Ubiquitination Pathway	9.31
mTOR Signaling	8.58
NF-κB Signaling	7.94
TNFR1 Signaling	7.63
Role of PKR in Interferon Induction and Antiviral Response	7.4
TNFR2 Signaling	7.19
TWEAK Signaling	7.03
Germ Cell-Sertoli Cell Junction Signaling	6.99
IL-8 Signaling	6.48
EIF2 Signaling	6.39

On Average, Over a Quarter of All PSGs Across Phenotypes Are DEGs in Patients

**Figure 3:**
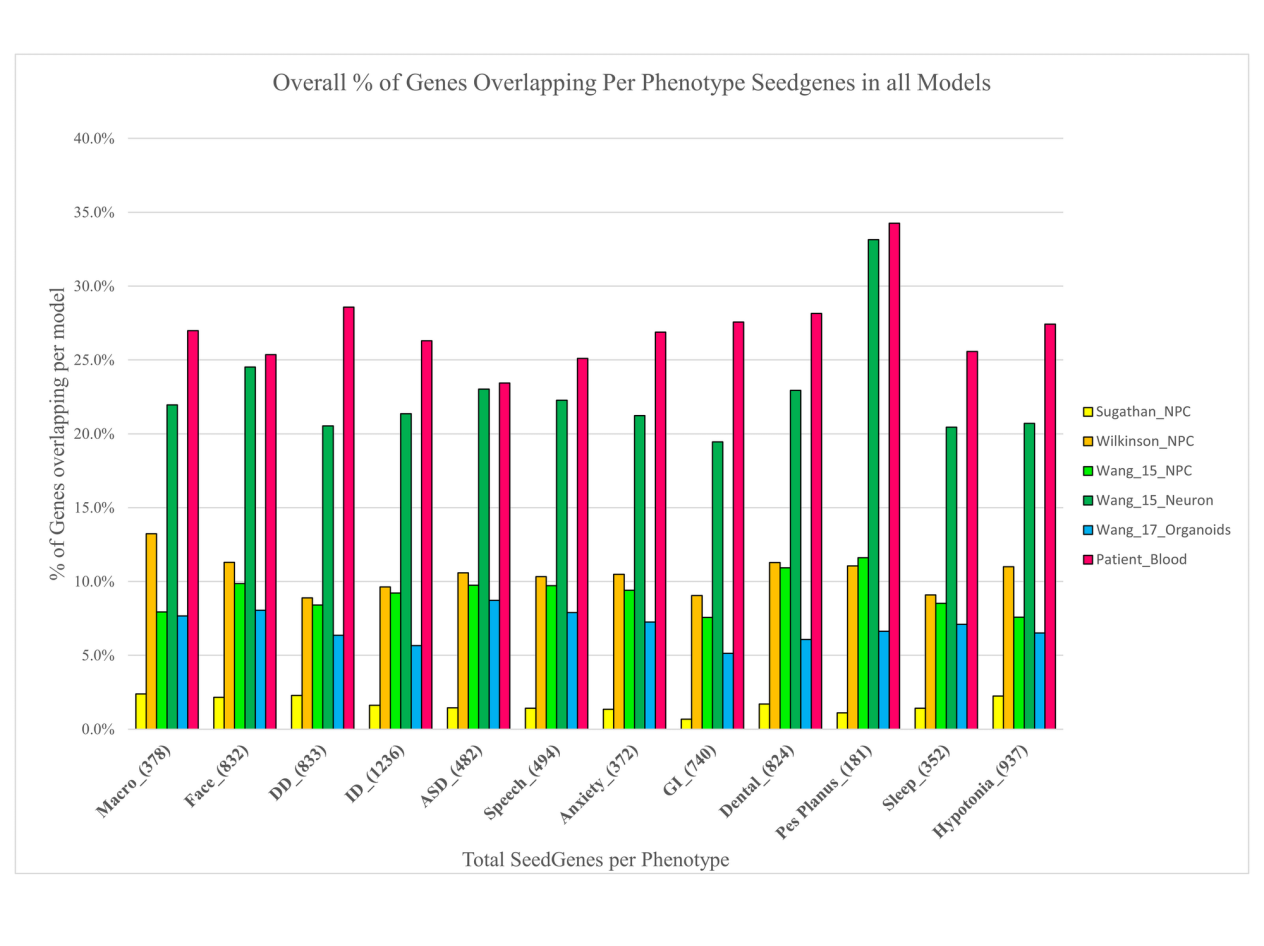
Bar plot showing the percentage of DEGs that appear as PSGs in each study, by phenotype DEGs: differentially expressed genes; PSGs: phenotype seed genes

We then sought to investigate the involvement of DEGs in possible phenotype-developmental pathophysiology by investigating which DEGs are PSGs. An average of 27.3% of genes from each phenotype seed gene list was found as a DEG in the patient (Table 3 and Figure 3), corresponding to an average of 3.2% of patient DEGs being included in each phenotype seed gene list. The highest number was found for the ID phenotype, having 327 patient DEGs that were also in this phenotype seed gene list (6% of the total patient DEGs). In total, 724 of the patient’s 5,388 DEGs were included as one or more PSGs. Appendix 3 contains tables that give a sample-based and phenotype-based summary of overlaps between DEGs and PSGs across all samples and phenotypes.

**Table 3:** Patient DEG overlap with PSG details DEG: differentially expressed gene; PSG: phenotype seed gene; DD: developmental delay; ID: intellectual disability; ASD: autism spectrum disorder; GI: gastrointestinal

Phenotype	Patient DEGs that are PSGs	Total PSG#	Percentage PSGs that are patient DEGs	Percentage of total patient DEGs (5388) that are PSGs
Facial dysmorphisms	212	832	25.50%	3.90%
DD	240	833	28.80%	4.50%
ID	327	1236	26.50%	6.10%
ASD	113	482	23.40%	2.10%
Speech delay	124	494	25.10%	2.30%
Macrocephaly	103	378	27.20%	1.90%
Pes planus	62	181	34.30%	1.20%
Hypotonia	258	937	27.50%	4.80%
GI	206	740	27.80%	3.80%
Sleep	90	352	25.60%	1.70%
Anxiety	101	372	27.20%	1.90%
Dental	235	824	28.50%	4.40%

Patient DEGs That Are PSGs for Multiple Phenotypes Include Well-Recognized ND Causative Genes

We looked for patient DEGs that appear in over one PSG list as possibly important candidates that may influence a wide spectrum of phenotype pathophysiology. Sixteen DEGs in the patient were PSGs in at least nine of the 12 phenotypes (Table 4). Of these, CDKN1C and MECP2 are PSGs in 11 out of the 12 phenotypes. CDKN1C appearing as a PSG in all but speech defects and MECP2 appearing as a PSG in all but pes_planus (Appendix 3). WBSCR27 is the most deregulated with a three-fold reduction in expression while CDKN1C is the next deregulated, with a one-fold increase in expression.

**Table 4:** Patient DEGs that are frequent PSGs DEGs: differentially expressed genes; PSGs: phenotype seed genes

Patient DEGs	Total times found out of the 12 phenotypes	Log2 (fold change)	Regulation
CDKN1C	11	1.10845	up
MECP2	11	0.37637	up
NFIX	10	0.413196	up
LIMK1	9	0.563392	up
FLNA	9	0.535682	up
BCL7B	9	0.419455	up
WBSCR16	9	0.30959	up
FLII	9	0.23419	up
COG6	9	-0.334932	down
NRAS	9	-0.34015	down
TCF4	9	-0.351494	down
ATRX	9	-0.377614	down
KRAS	9	-0.427387	down
PTEN	9	-0.524188	down
DHFR	9	-0.565876	down
WBSCR27	9	-3.16821	down

Comparison of the patient transcriptome with cellular model transcriptomes

The DEG landscape across the patient sample and all models is discordant, with only FBXL19 and EML6 appearing as DEGs in all datasets: We compared the number and distribution of DEGs across all cellular samples and the patient’s transcriptome. DEGs that met the expression threshold of q>0.5 in all models and in the patient are given in Appendix 3. The most DEGs were found in the patient (5,388) while only considering the cellular models, the most DEGs were identified by Wang et al. in neuron cells (3289). Sugathan et al. had the least (369) in NPCs. On average, 2,157 DEGs were found across all samples, with a median number of 1,530 DEGs (Table 5).

**Table 5:** Total DEG counts DEG: differentially expressed gene

Model	Number of DEGs
Patient blood	5388
Neurons_ Wang 2015	3289
NPC_ Wilkinson	1812
NPC_ Wang 2015	1248
Organoids_ Wang 2017	838
NPC_ Sugathan	369
DEGs Average	2157
DEGs Median	1530

We sought to determine the overlap among the DEGs identified across all datasets. Concordance was poor among all transcriptomes, with only FBXL19 and EML6 as concordant DEGs across all datasets. Omitting Sugathan et al., we found CELF2, PODXL, RGMB, NFIA, and TCF4 were differentially expressed in the four cellular model datasets and in the patient transcriptome. There are no common DEGs to all the five cellular transcriptomes that are not found in the patient. RIMS3 was the only other gene common to four cellular models and the patient; it was not a DEG only in the Wang organoid sample. Of the eight genes above, we note that FBXL19 and EML6, as well as CELF2, PODXL, and RIMS3, are not seed genes in any of our PSG lists while TCF4, NFIA, and RGMB are PSGs (Table 6).

**Table 6:** DEGs that are PSGs and found in at least five of the six samples, arranged according to phenotype DEGs: differentially expressed genes; PSGs: phenotype seed genes; DD: developmental delay; ID: intellectual disability; ASD: autism spectrum disorder; GI: gastrointestinal

	Macro	Face	DD	ID	ASD	Speech	Anxiety	GI	Dental	PP	Sleep	Hypotonia	Non-phenotype
Patient		TCF4	TCF4	TCF4	TCF4 RGMB	TCF4	NFIA	TCF4 NFIA	TCF4	TCF4		TCF4 NFIA	CELF2, EML6, FBXL19, PODXL, RIMS3
Wang Organoid		TCF4	TCF4	TCF4	TCF4 RGMB	TCF4	NFIA	TCF4 NFIA	TCF4	TCF4		TCF4 NFIA	CELF2, EML6, FBXL19, PODXL
Wang Neurons		TCF4	TCF4	TCF4	TCF4 RGMB	TCF4	NFIA	TCF4 NFIA	TCF4	TCF4		TCF4 NFIA	CELF2, EML6, FBXL19, PODXL, RIMS3
Wang NPC		TCF4	TCF4	TCF4	TCF4 RGMB	TCF4	NFIA	TCF4 NFIA	TCF4	TCF4		TCF4 NFIA	CELF2, EML6, FBXL19, PODXL, RIMS3
Wilkinson		TCF4	TCF4	TCF4	TCF4 RGMB	TCF4	NFIA	TCF4 NFIA	TCF4	TCF4		TCF4 NFIA	CELF2, EML6, FBXL19, PODXL, RIMS3
Sugathan													EML6, FBXL19, RIMS3

The rate of overlap between PSGs and DEGs differs among the datasets: We then conducted a focused sub-analysis, looking only at DEGs that are also PSGs. As with the overall overlap of DEGs among the RNAseq dataset, we found similar discordance when looking at those DEGs that were also PSGs. The highest percentage of phenotype seed gene involvement was found in the patient’s RNAseq data while the lowest was from the Sugathan et al. dataset. However, we note the Wang neuron transcriptome showed a distribution most resembling that of the patient, with an average of 22.3% for the 12 PSG lists found as Wang neuron DEGs (Figure 3).

TCF4, NFIA, and RGMB are differentially expressed PSGs common across the patient transcriptome and four of the five cellular model transcriptomes: There was no PSG that appeared as DEGs in all six transcriptome datasets. However, three PSGs - TCF4, NFIA, and RGMB - appear as DEGs in the patient and in four cellular transcriptomes, i.e., in all except the Sugathan dataset (Table 6 and Appendix 5). TCF4 is a phenotype seed gene for DD, ID, ASD, speech delay, GI problems, dental, Pes planus, hypotonia, and facial dysmorphisms; NFIA is a phenotype seed gene for anxiety, GI problems, and hypotonia; and RGMB appears as a phenotype seed gene for ASD.

## Discussion

Patient transcriptome shows widespread gene deregulation and affects key pathways

Almost 50% of detected genes were differentially expressed in the patient versus his normal brother, a finding in keeping with other studies in models [4-7]. Thus, our finding further corroborates the growing evidence that CHD8 has widespread downstream targeting [8].

The fact that approximately half of all detected genes are differentially expressed, and that several dozen genes show over a three-fold expression change prompted us to conduct pathway analyses as an informative data interpretation approach. IPA analysis revealed the UPP to be the most altered in expression. The UPP is an important pathway in sculpting the developing nervous system, maintaining brain plasticity in response to environmental stimuli, and has been implicated in the causation of ND [9]. The UPP is implicated in the development of both pediatric and adult brain tumors [10]. Importantly, the link between the UPP and ASD has also been gaining attention [11]. Our results draw further attention to this pathway as important in ND across diagnostic boundaries.

The second most deregulated pathway is the mTOR signaling pathway. Indeed, it and the UPP are elevated in comparison to the next eight most deregulated pathways that cluster together (Table 2). The mTOR pathway is an upstream regulator of WNT signaling, which has been reported by a number of model studies in CHD8+/- and Chd8+/- to be among the topmost perturbed [4-7].

Meta-analyses across all samples show poor concordance

**Figure 4:**
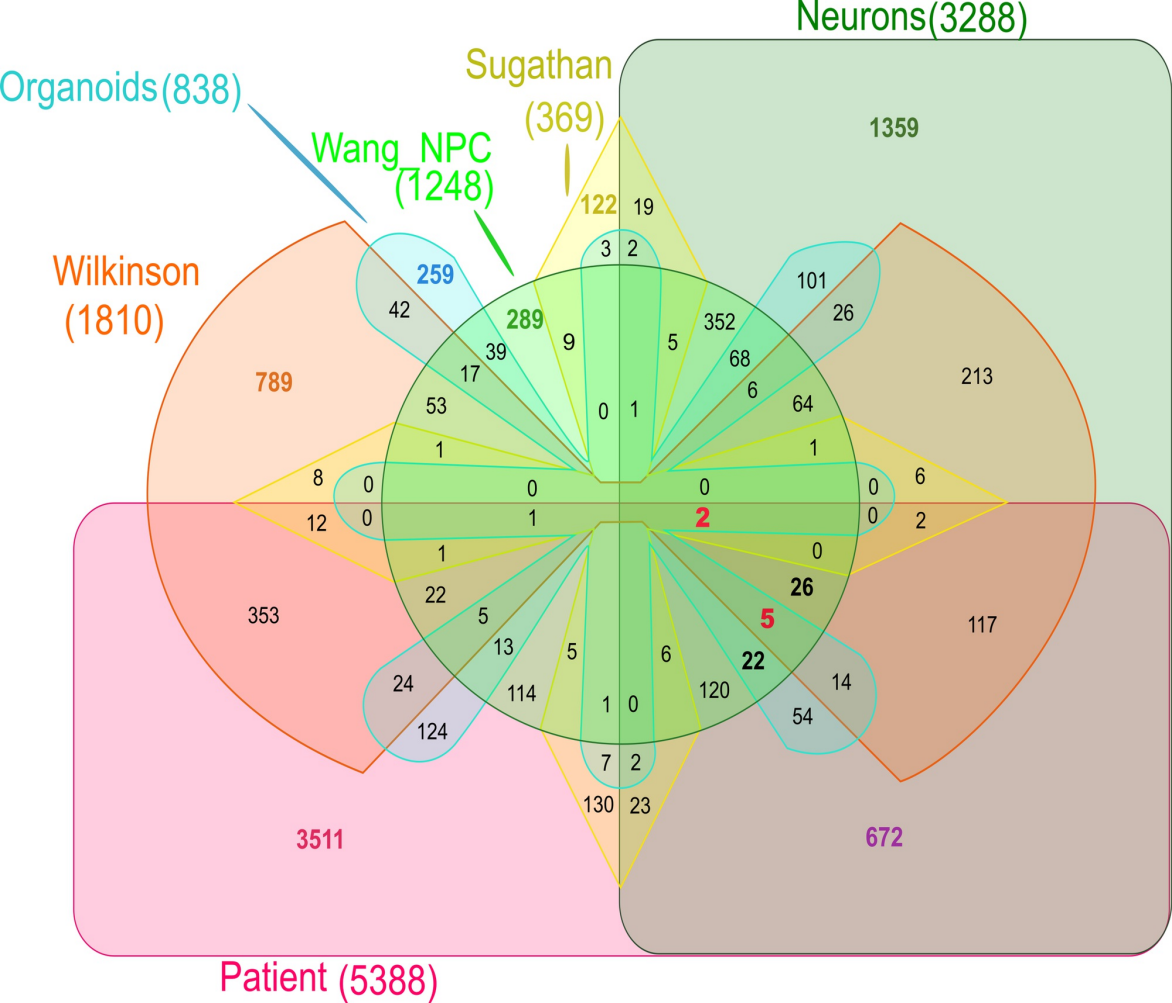
Six-way Venn diagram showing an overlap between all DEGs across the six studies DEGs: differentially expressed genes

When considering all our transcriptome profiles together, only two genes, EML6 and FBXL19, appeared as DEGs across all six sample transcriptomes, exemplifying overall poor concordance (Figure 4). Differences in the sample source material, laboratory, and bioinformatic methods are among possible reasons for the discordance as we discuss them under the study limitations. Nevertheless, several genes with important functions are found commonly differentially expressed across samples (Figure 4 and Table 6). We discuss them briefly later in this section.

Patient’s blood transcriptome DEG profile most resembled that of the neuronal cell model

Remarkably, the DEG profile of the neuronal model, and not that of NPCs, nor the organoid, most resembled that of our patient’s transcriptome. The neuronal model produced the highest number of DEGs next to the patient transcriptome; 1,063 DEGs are common between the two datasets, of which, 672 were only found in only the patient and the neuronal transcriptome (Figure 4). Blood cells and neurons arise from different germ layers, the mesoderm, and ectoderm, respectively. Yet, consistent with our finding, a high degree of similarity is observed between the transcriptomes of blood and brain, with estimates of 35% to 80% of known transcripts to be present in both tissues and correlation in expression levels up to 0.64 observed [12]. In contrast, the lower concordance between blood and organoid transcriptome is surprising. While the complex differentiation signals undergone by neurons during organoid generation may play a part, we suggest this finding be explored further, as possible explanatory information is sparse.

Syndrome-phenotype informed DEG filtration yields key candidate genes

A key strength of our study is our informed phenotype-based data filtration approach. Having extensively cataloged the phenotypic spectrum of all reported ZFS patients [2], we utilized this knowledge as a means to identify possible key CHD8 targets. Twelve phenotypes are most commonly presented by ZFS patients as given in this paper’s introduction (see Appendix 2 for the full list).

CHD8 is an epigenomic regulator [3] and haploinsufficiency is the causative mechanism for ZFS syndrome [2]. Genes already considered candidates for phenotypes in ZFS presentation, which we find to be significantly deregulated in either the patient or the cellular models, are therefore hypothesized to be good candidates for further study of ZFS manifestation.

Across the six transcriptomes and 12 phenotypes, the highest proportion of PSGs that were found as differentially expressed genes were found in the patient and then in the Wang neuronal sample. On average, a quarter of the PSG per phenotype was found in the DEGs of the patient transcriptome (Figure 3).

In the patient, the most upregulated and most downregulated PSGs found differentially expressed (Table 4) are both known to be causative for ND:

CDKN1C (cyclin-dependent kinase inhibitor 1C) encodes a tumor suppressor involved in growth control. It is located near the IC2 imprinting center. Reduction in the activity of CDKN1C is implicated in Beckwith-Wiedemann syndrome, an overgrowth syndrome. Conversely, maternal uni-parental isodisomy resulting in over-expression (as the maternal allele is hypomethylated) has been shown to cause IMAGe syndrome - a rare disease that includes intrauterine growth restriction, metaphyseal dysplasia, adrenal hypoplasia congenita, and genital anomalies [13]. In our patient, this gene had a one-fold increased expression as compared to the control. It is a PSG in all 12 phenotypes except for speech defects.

WBSCR27, also known as METTL27, is a gene located in a contiguous gene deletion region at chromosome 7q11.22-q11.23 considered to be causative for Williams-Beuren syndrome, a rare autosomal dominant ID syndrome that includes multi-system phenotypes and characteristic facies. WBSCR27 encodes a methyltransferase belonging to the ubiE/CoQ5 family. Curiously, none of the cellular models report WBSCR27 as differentially expressed (Appendix 3). WBSCR27 had a three-fold reduction of expression level in the patient. This gene is a PSG in all phenotypes except for macrocephaly, DD, and speech defects (Appendix 3).

PSG involvement across all samples highlights potential lead pathophysiology target genes with a role in multiple phenotypes

When we looked at PSG involvement across all samples (see Appendix 5 (A to L) for Venn diagrams of overlap per each phenotype), we were able to highlight a few genes that have high recurrence both among samples as well as among phenotypes (Table 6).

Only three PSGs - TCF4, NFIA, and RGMB- are differentially expressed in the patient and at least four other models. TCF4 is a PSG in nine of the 12 phenotypes, NFIA is a PSG in three, and RGMB is a PSG for only ASD (Table 6). We discuss them briefly below.

TCF4 (transcription factor 4) encodes a member of the basic helix-loop-helix (bHLH) protein family that regulates transcription by binding promoters and enhancers. TCF4 appears to be a master driver for neurodevelopment, with demonstrated wide-range target-binding [14]. Common variants associated with schizophrenia and corneal endothelial dystrophy risk, and rare mutations causing the autosomal dominant Pitt-Hopkins syndrome [15] are reported. TCF4 has been well-studied as a regulator of neural differentiation and has known important functions in pathways associated with NDs, including ASD and ID [14]. Several features characteristic of Pitt-Hopkins syndrome are also found in our patient; DD, fine motor skills, gastrointestinal problems, and speech problems are common [2]. Emerging evidence shows TCF4 expression is a targetable prognostic indicator in cancer [16]. The TCF4 gene appears in all models excluding Sugathan et al. as a DEG and is a PSG in a remarkable nine of our 12 phenotypes (Table 6). These data and the known wide range of TCF4 involvement in cellular programming support the notion that a CHD8 haploinsufficiency-induced alteration in TCF4 expression is a key molecular signal in the pathophysiology of this condition.

RGMB (repulsive guidance molecule family member B) encodes a protein called DRAGON that is part of the repulsive guidance molecule (RGM) family of proteins. RGMs have been shown to play a role in the development and regulation of nervous and skeletal tissue and the gastrointestinal and immune system [17]. Oncogenic activity [18] and function during gut development for the mouse ortholog, as well as the modulatory activity for neurite outgrowth following spinal cord injury in rat [17], has been reported. We note the importance of DRAGON in fine control of brain homeostasis and its involvement in the gut as remarkable in ASD pathophysiology for which it is a PSG.

NFIA (Nuclear Factor I A) is one of the four-member nuclear factor 1 (NFI) transcription factors, that have important functions in the development of the brain among other organs. An autosomal dominant NFIA-related disorder with corpus callosum abnormalities and macrocephaly is reported, and it has been suggested to be the critical gene for the chromosome 1p32-p31 deletion syndrome [19].

In summary, our ZFS presentation informed data filtration approach conducted across these diverse samples has succeeded in drawing a spotlight onto what may be the key targets of CHD8 in disease pathophysiology for this syndrome. Further studies are warranted to determine the impact of the above genes on ZFS.

Commonly differentially expressed genes that are not found as PSGs

Recognizing that the definition of a PSG is entirely dependent on the corpus of accumulated data on gene function and pathology, we also carried out filtration for genes commonly differentially expressed across all samples that are not in any of the PSG candidates (Table 6). Two genes - EML6 and FBXL19 - were significantly differentially expressed in all six samples while two more -CELF2 and PODXL - were significantly differentially expressed in five samples excluding the low-yielding Sugathan transcriptome. Another gene, RIMS3 is notable for being differentially expressed in all samples except the organoid sample. We briefly discuss these genes below.

DEGs Common to All Six Samples

EML6 (echinoderm microtubule-associated protein-like 6) is a protein-coding gene of which not much is known. EML6 was found to localize on microtubules and showed high expression in E11 mouse embryos while declining in adults, which makes it a highly developmentally regulated gene that may be modifying the assembly dynamics of microtubules [20], precluding further comment.

FBXL19 (F-box and leucine-rich repeat protein 19) encodes a protein member of the E3 ubiquitin ligases. Several studies report that FBXL19 and its anti-sense mRNA, FBXL19-AS1, have important roles in various cancers [21]. In mice, it was recently shown that Fbxl19 is a CpG island-binding protein, enabling gene activation during cell lineage commitment, and is essential for development [22]. Thus, while FBXL19 was not selected as a PSG, its activity indicates an important, though as yet undefined, role in phenotype pathophysiology.

DEGs Common to All Samples Except the Sugathan Sample

CELF2 (CUGBP Elav-like family member 2) is a tumor suppressor that encodes CELF2, a protein that is one of the six-member CELF family of proteins that are recognized for their wide-ranging function in regulating pre-mRNA splicing and controlling the translation and stability of mRNAs [23]. While other family members have a more specific expression, CELF2 and CELF1 are broadly expressed [23]. There is strong evidence that CELF2 plays a role in myotonic dystrophy in particular and neurological disease in general [23]. CELF2 is a tumor suppressor with activity in cancers reported [24]. These data argue in favor of a possible wide-ranging influence for this gene on the pathophysiology of the syndrome. We particularly note this gene’s involvement in motor function and consider this important with respect to the motor phenotypes seen in the syndrome, though it is not an identified PSG.

PODXL (podocalyxin-like) encodes a neural adhesion molecule and has been shown to cause autosomal recessive juvenile Parkinsonism [25]. A causative role for nephrosis and indicative roles in glomerular diseases [26], as well as a prognostic factor for some types of cancer [27], are known.

DEG Common to All Except the Organoid Sample

RIMS3 (regulating synaptic membrane exocytosis 3) encodes a protein thought to function in regulating synaptic membrane exocytosis. The fact that it does not appear as a DEG in the organoid sample but does so in all other samples likely indicates it is turned off later in differentiation.

In summary, this filtration has focused attention on candidate genes that require further study as important downstream targets of CHD8 in ZFS etiology. Additionally, looking at Table 6, we are able to obtain a preliminary insight into a potential gene expression cascade as we see DEGs that are common to samples spanning varied developmental time-points as well as specific phenotypes.

Common DEGs Are Also Cancer Susceptibility Genes

As noted above, many of these genes are cancer susceptibility genes. We previously noted the link to cancer for CHD8 haploinsufficiency, highlighting that the father of a patient with a paternally inherited CHD8 sequence mutation, who also had similar autistic features to the patient, developed cT2N1 rectum carcinoma [2]. Here, our findings of key DEGs that have wide-ranging involvement in both neurodevelopment and cancer further serve to underline the importance of cancer screening for patients with CHD8 haploinsufficiency.

Study strengths and weaknesses

While we have highlighted several interesting genes that may play a significant role in ZFS causation in this study, we caution that this is a preliminary investigation and more work is required to make stronger conclusions. We are limited by the number of patient samples used. We were uncertain whether using patient blood samples would allow us to meaningfully investigate the gene expression profile for a neurodevelopmental disorder. Therefore, taking this first patient’s blood transcriptome, we compared it to five different human cellular models and were able to show that the patient profile most resembles a neuronal profile, leading the way for more patient blood transcriptomes to be profiled.

We note the variety of sample material used may confound results. The six transcriptome datasets include our patient’s venous blood-derived PBMC sample, three NPC samples from different groups [4-6], a neuronal cell sample, and an organoid sample from the same group [6-7] (Appendix 1). Yet, among the three datasets that are all from NPCs, the data substantially differ (Figure 4), indicating a sample type bias may not play as much of a role as thought. Further having this variety in the developmental stage allowed us to unearth candidate genes important across developmental stages (Table 6).

We acknowledge there is a wide variety of experimental and bioinformatic methods used across all studies (Appendix 1). Remarkable differences include the advanced donor age in the Sugathan study (cells were derived from a 63-year-old human control subject). Donor age has been shown many times to impact reprogramming efficiency in mice [28] and may explain the markedly different results we obtained from the Sugathan sample profile. Also noteworthy is the fact that the Wang et al. datasets did not include CHD8 as significantly differentially expressed. They could not give an exact reason for this (personal communication). However, the CHD8 expression level reported by Wang is most close to a fold change of 0.5 in their neuronal model versus their NPC and their organoid models [7]. This explains why CHD8 does not appear as a common overlapping gene among all datasets for phenotypes such as ID, ASD, and speech delay, for which it is a well-documented known gene, as would be expected.

We were concerned that differences in bioinformatics data processing among the studies could confound results. However, Wade et al., who analyzed a larger series of publicly available datasets, including the five we used, and who subjected the raw sequence data to a uniform in-house bioinformatic pipeline, still report discordancy [8].

Taking these potential causes for variation into consideration, we reasoned that since uniform bioinformatic processing does not alleviate discordance and since we cannot cancel out the sample and experiment specific-bias inherent to each study, we would accept such study-dependent inherent bias and instead apply a uniform differential expression threshold of q>0.5 to define DEGs to be used in our meta-analysis, so that our acceptance threshold for DEGs would alleviate between study bias as much as possible.

Finally, we surmised that conducting the phenotype-informed focused analysis is a means to derive meaningful concordant data, as demonstrated by our findings. However, as with all exploratory analyses, we cannot rule out there could be other important target genes missed by our approach.

## Conclusions

Here we report the first whole transcriptome RNAseq analyses for a patient with ZFS. Comparing our patient transcriptome to five, different, human cellular engineered model transcriptomes, using both a non-informed and patient phenotype-informed analysis, we are able to unearth key biological pathways and downstream target genes that include those with known phenotypic involvement, as well as novel targets. We show that CHD8 haploinsufficiency in the patient causes widespread transcriptome expression change and affects a number of molecular pathways, as has been reported in both cellular and animal models. By examining the comparison between our patient transcriptome and cellular models that included neuronal progenitors, neurons, and brain-organoids, we strikingly discover that the patient blood transcriptome most resembles a neuronal cellular model. This finding is encouraging for further transcriptome profiling of patient blood samples as a means to profile this ND condition.

In summary, in this preliminary observational analysis, we are able to show patient blood transcriptomes are a viable option for further gene expression profiling of CHD8+/- impact on ND, and highlight important genes with both known and novel involvement with ZFS phenotypes that may potentially be key players in syndrome pathophysiology.

## Figures and Tables

**Figure 5:**
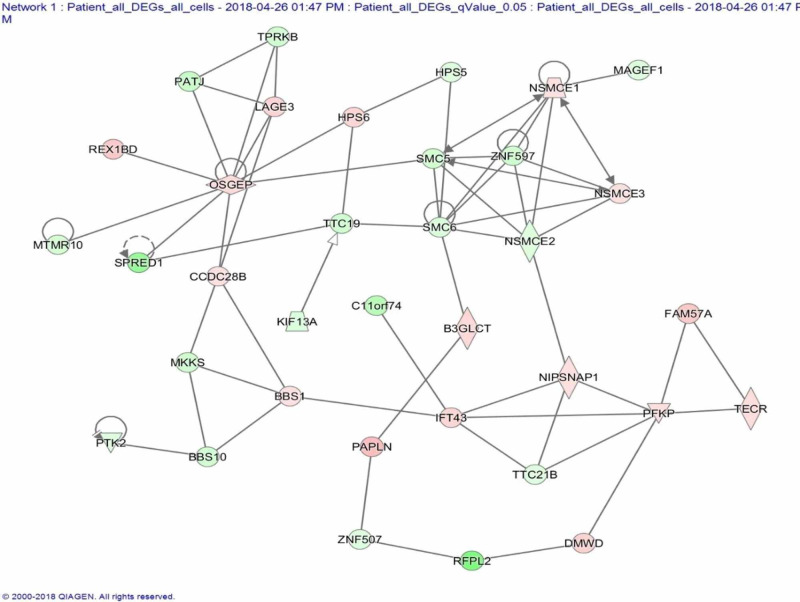
Connections and regulation of molecules found in the most important network “Developmental Disorder, Hereditary Disorder, Neurological Disease” from the patient’s RNAseq data

**Figure 6:**
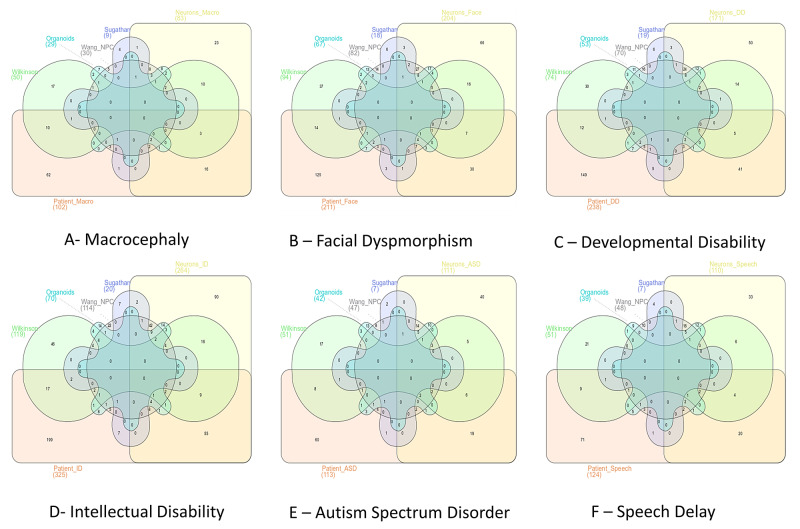
Part A - six-way Venn intersections of DEGs across samples according to each phenotype DEGs: differentially expressed genes

**Figure 7:**
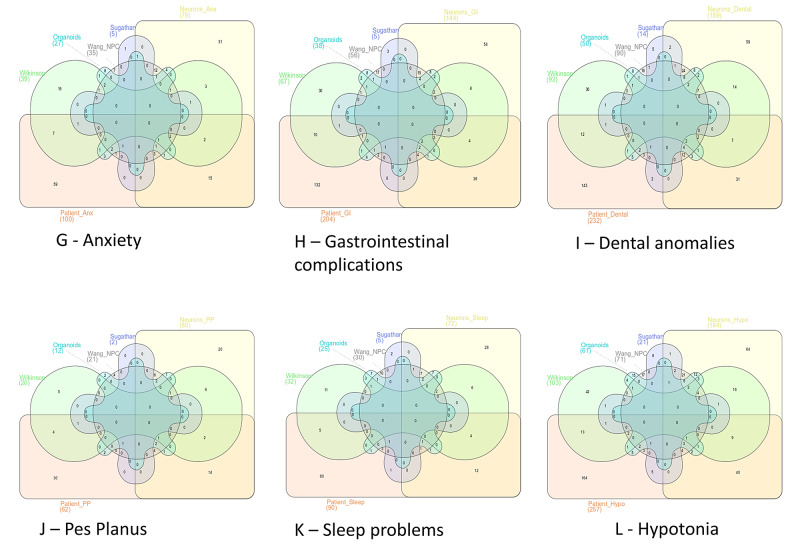
Part B - six-way Venn intersections of DEGs across samples according to each phenotype DEGs: differentially expressed genes

## Appendices

Appendix 1

A table giving an overview of laboratory methods and bioinformatics pipelines used for each transcriptome dataset:

**Table 7:** An overview of laboratory methods and bioinformatics pipelines used by each dataset presented in this paper

Dataset	Patient	Sugathan NPCs	Wilkinson NPCs	Wang NPCs and Neurons	Wang Organoids
GEO accession no.		GSE61492		GSE71594	GSE85417
Cell type	PBMCs	Induced pluripotent stem cells (iPSCs) derived neural progenitor cells (NPCs) from human control (GM8330)	Commercial (SK-N-SH) human NPCs from neuroblastoma cells	iPSCs and neurons developed from human control fibroblasts obtained from a skin biopsy.	Differentiated cerebral organoids derived from developed iPSC-derived NPCs from Wang et al., 2015.
Samples	3 samples 3 controls	14 samples 8 controls	4 samples 4 controls	NPCs: 2 samples, 2 controls. Neurons: 2 samples, 2 controls.	4 samples 2 controls
Gene knockdown method	Native	Transduction by HIV-based lentiviral vectors carrying shRNAs targeting the CHD8 gene.	Transfection by siRNA silencer selective negative control and siRNA targeting the CHD8 gene	Transduction with CRISPR/Cas9 vectors containing two separate CRISPR sgRNA sequences to target the N-terminal of CHD8 protein to generate truncated mutations	
NGS	Total RNAseq	Strand-specific dUTP RNAseq. 40.6 million reads per line at a final depth ~45 million for total reads.	RNAseq focusing on long noncoding RNAs. Average of 45 million per knockdown replicate and an average of 40 million reads per control replicate.	Total RNAseq	Total RNAseq
Number of DEGs	5388	369	1812	NPCs: 1248, Neurons: 3289	838
Mapping/ alignment:		Gsnap (GrCH37) ensemble (71)	transcriptome :UCSC Hg19,Ensemble GrCH37 (75),Tophat2	Tophat (hg19) Gencode database, Cufflink used to get FPKM	Kallisto using GENCODE. TPM > 1 in WT samples
DE analysis		DESeq (2 factor model) 1.12.1	Cuffdiff + GTF file of the transcriptome using default settings	DESeq2	DESeq2
Functional analysis		DAVID, ToppGene	DAVID, Disease association protein-protein link evaluator	DAVID, IPA, ToppGene	DAVID, IPA

Appendix 2

Notes describing (A) Phenotype seed gene list generation parameters and (B) IPA pathway analysis settings used in this study.

(A) Phenotype Seed Gene List Generation

The following search terms were entered into Phenolyzer (http://phenolyzer.wglab.org/) in order to obtain seed genes for each phenotype.

Phenotype 1: Facial dysmorphisms. The following unique terms were searched, and results amalgamated as PSGs for ‘facial dysmorphisms’: “auricular malformation”, “cupid bow upper lip”, “cupid bow shaped upper lip”, “high palate”, “hypertelorism”, “long philtrum”, “prominent supraorbital ridges”, “down slanting palpebral fissure”, “down slanted palpebral fissures”, “downward slanting palpebral fissures”, “downward slanted palpebral fissures”.

Phenotype 2: Behavioral presentations: “anxiety”.

Phenotype 3: DD: “developmental delay”.

Phenotype 4: ID: “intellectual disabilities”.

Phenotype 5: ASD: “autism”, “autistic”, “autism spectrum disorders”.

Phenotype 6: Macrocephaly: “Macrocephaly”.

Phenotype 7: Speech complications: "speech delay”, “language development disorder". Phenotype 8: Skeletal complications: “pes planus”.

Phenotype 9: Neurological complications: “hypotonia”.

Phenotype 10: GI complications: "diarrhea”, “constipation".

Phenotype 11: Sleep complications: “sleep disturbance”, “sleep disorder".

Phenotype 12: Dental complications: "dental anomalies"

(B) IPA Pathway Analyses:

Analysis creation date: 2018-04-26 and build version: 470319M.

Analysis settings:

·         Reference set: Ingenuity Knowledge Base (Genes Only)

·         Relationship to include: Direct and Indirect

·         Includes Endogenous Chemicals

·         Filter Summary:

Consider only relationships where (confidence = High (predicted) OR Experimentally Observed) AND (data sources = An Open Access Database of Genome-wide Association Results OR BIND OR BioGRID OR ClinicalTrials.gov OR ClinVar OR Cognia OR DIP OR DrugBank OR Gene Ontology (GO) OR GVK Biosciences OR Hazardous Substances Data Bank (HSDB) OR HumanCyc OR Ingenuity Expert Findings OR Ingenuity ExpertAssist Findings OR IntAct OR Interactome studies OR MIPS OR miRBase OR miRecords OR Mouse Genome Database (MGD) OR Obesity Gene Map Database OR Online Mendelian Inheritance in Man (OMIM) OR TarBase OR TargetScan Human)

Appendix 3

Below is a list of tables and datasets that are too large to be appended. They are available upon request. 

(A) Table of all phenotype seed genes organized by phenotype - 18-page PDF document.

(B) Table of all genes found expressed by RNAseq in the patient with log2fold change values and measures of statistical significance - 573-page PDF document.

(C) Table of all differentially expressed genes in the six transcriptome samples organized by sample - 69-page PDF document.

(D) Multi-sheet Excel workbook containing a summary of overlaps between DEGs and PSGs organized by sample.

(E) Multi-sheet Excel workbook containing a summary of overlaps between DEGs and PSGs organized by phenotype.

Appendix 4

Pathway diagram for the most important network “Developmental Disorder, Hereditary Disorder, Neurological Disease” from the patient’s RNAseq data

Appendix 5

This Appendix contains two images (to maintain readability), which contain 12 panels, each depicting a six-way Venn diagram for intersections of all DEGs among samples by phenotype. Part A - panels A to F and Part B - panels G to L.
